# Association between neutrophil percentage to albumin ratio and sarcopenia among cancer patients: evidence from both the Chinese and American cohorts

**DOI:** 10.3389/fnut.2025.1709323

**Published:** 2026-01-20

**Authors:** Yang Yu, Bo Chen

**Affiliations:** Department of General Surgery, The First Affiliated Hospital of Anhui Medical University, Hefei, China

**Keywords:** cancer, mediation analyses, multicenter study, NPAR, sarcopenia

## Abstract

**Background:**

Sarcopenia, the progressive depletion of skeletal muscle mass and strength, worsens quality of life, and survival in cancer patients. Given its multifactorial pathogenesis involving chronic inflammation and malnutrition, integrated biomarkers for early risk assessment are needed.

**Methods:**

This two-cohort study investigated the clinical utility of the neutrophil percentage-to-albumin ratio (NPAR) for sarcopenia risk stratification. We analyzed data from the US National Health and Nutrition Examination Survey (NHANES, *n* = 1,586) and a Chinese clinical cohort (*n* = 705). Associations were assessed using multivariable regression, threshold analysis, and ROC curves. Mediation analyses were performed in the NHANES cohort.

**Results:**

Elevated NPAR was significantly associated with higher odds of sarcopenia in both the American (OR = 1.14, 95% CI: 1.06–1.22) and Chinese (OR = 1.10, 95% CI: 1.04–1.14) populations, showing a dose-response relationship. Non-linear threshold effects were identified at NPAR = 14.7 (NHANES) and NPAR = 17.07 (Chinese cohort). NPAR outperformed other inflammatory indices in the clinical cohort (AUC = 0.568). Systemic inflammation (C-reactive protein) and dietary protein intake mediated 17.10% and 6.37% of the association, respectively. Sensitivity analyses supported robustness.

**Conclusions:**

NPAR is a practical and cost-effective inflammatory-nutritional biomarker for sarcopenia risk stratification in cancer patients. It shows promise for early identification and personalized intervention across diverse populations.

## 1 Introduction

Sarcopenia, characterized by the progressive loss of skeletal muscle mass and function, is a common complication among cancer patients and significantly impacts their quality of life, treatment tolerance, and survival outcomes (1–6). The pathogenesis of sarcopenia in cancer patients is multifactorial, involving factors such as cancer-related inflammation, altered metabolism, and nutritional deficiencies (7–9). Developing validated biomarkers for early-phase detection and longitudinal monitoring of sarcopenia is imperative to implement precision clinical protocols through targeted interventions, thereby enhancing functional preservation in at-risk populations.

In recent years, the neutrophil percentage-to-albumin ratio (NPAR) has emerged as a promising biomarker for assessing inflammation and nutritional status (10, 11). NPAR is calculated by dividing the percentage of neutrophils by the serum albumin level and has been shown to be associated with various clinical conditions, including cardiovascular diseases, diabetes, Ulcerative Colitis, and liver diseases (12–15). NPAR's prognostic value in sarcopenia has garnered growing interest through its dual quantification of inflammatory dysregulation and nutritional depletion–recognized pathophysiological drivers of this condition (16).

Emerging research has systematically investigated NPAR correlations with diverse clinical endpoints. For instance, Ding et al. (17) demonstrated elevated NPAR levels served as an independent predictor for rheumatoid arthritis incidence in cohort studies. Similarly, Li et al. (18) reported that NPAR was associated with chronic kidney disease, emphasizing its prognostic value. Additionally, Wang et al. (19) demonstrated that NPAR was associated with nonalcoholic fatty liver disease, further supporting its utility as a biomarker for metabolic and inflammatory disorders.

Despite these findings, the relationship between NPAR and sarcopenia among cancer patients remains largely unexplored. Given the significant impact of sarcopenia on cancer patients' prognosis and the potential of NPAR to reflect both inflammation and nutritional status, investigating this association could provide valuable insights for early identification and intervention. We posit that NPAR holds particular promise as a composite reflector of the core pathophysiology. In contrast to tools that rely on subjective reporting (e.g., SARC-F) or are resource-intensive (e.g., full AWGS/EWGSOP assessment), NPAR is derived objectively from routine blood tests, offering a practical “first-line” signal for muscle wasting risk. The selection of NPAR is grounded in the multifactorial pathogenesis of cancer-related sarcopenia, which is driven by the synergistic effects of chronic inflammation and nutritional depletion. An elevated NPAR, irrespective of its primary driver (neutrophilia or hypoalbuminemia), provides an integrated signal of the pro-catabolic and anti-anabolic milieu that predisposes to muscle wasting.

Therefore, the present study aims to explore the association between NPAR and sarcopenia among cancer patients. This study enhances sarcopenia biomarker research by elucidating NPAR's potential as a clinical indicator in cancer populations, particularly addressing chemotherapy-associated metabolic dysregulation that may exacerbate muscle wasting processes.

## 2 Methods

### 2.1 Survey description

The analysis employed data from the NHANES, a nationally representative cross-sectional surveillance system administered by the CDC's National Center for Health Statistics (NCHS). Utilizing a stratified multistage probability sampling design (20), NHANES integrates demographic interviews, physical assessments, and laboratory analyses to monitor health parameters in community-dwelling Americans. This study incorporated data from discontinuous cycles spanning 1999–2006 and 2011–2018, excluding interim years to align with Dual-energy X-ray Absorptiometry (DXA) availability protocols (20, 21). More detailed information on the DXA examination protocol is documented in the NHANES Body Composition Procedures Manual. To externally validate our findings, we supplemented this with a clinical cohort of gastrointestinal cancer patients (2015–2020) from the Department of Gastrointestinal Surgery, the First Affiliated Hospital of Anhui Medical University.

### 2.2 Study population

**Figure 1:**
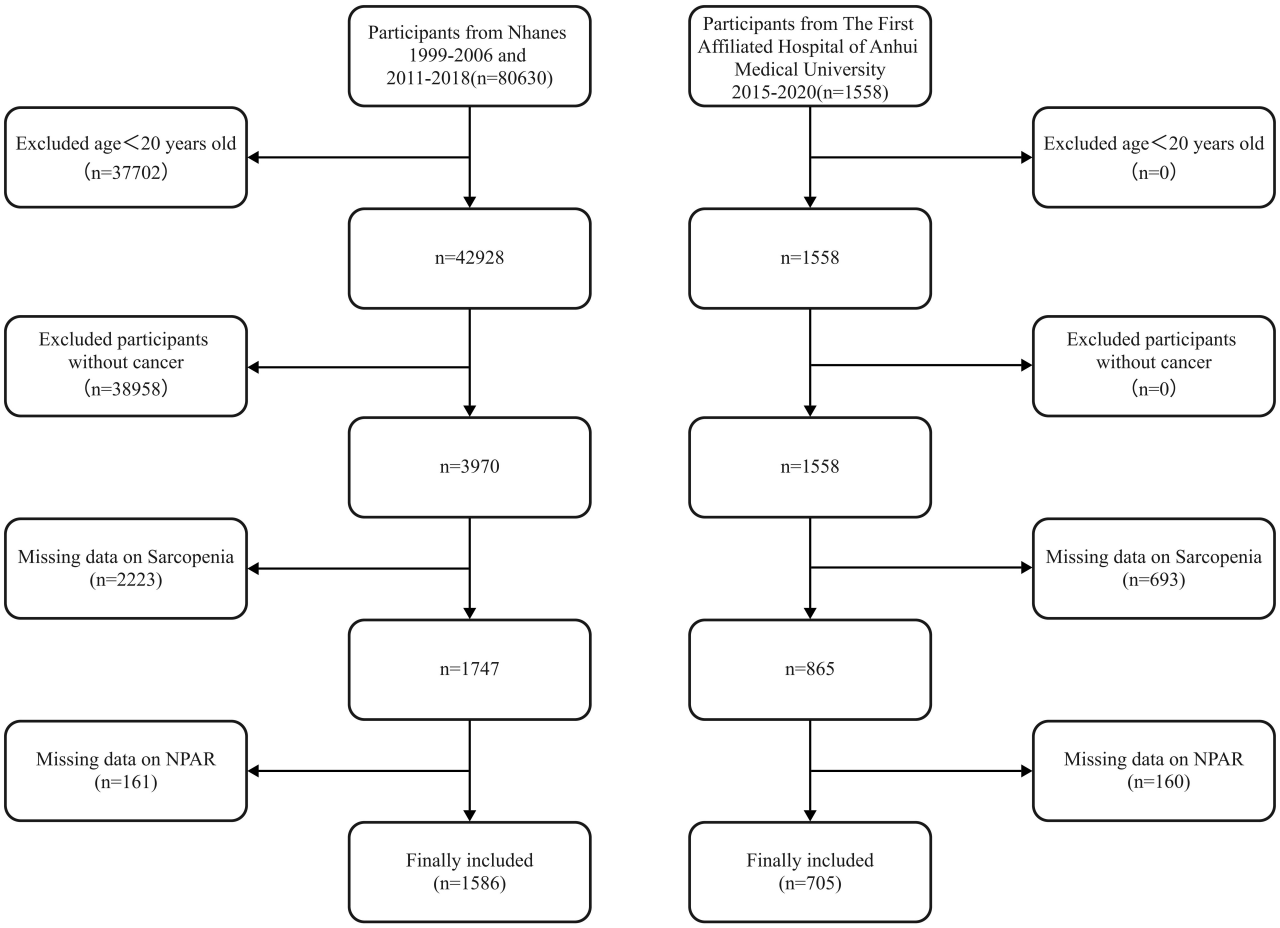
Flow chart of participant selection. This figure details the participant selection process for this bicontinental study. The **left panel** shows the derivation of the US cohort from the National Health and Nutrition Examination Survey (NHANES, 1999–2006 and 2011–2018), starting with 80,630 participants. The **right panel** shows the derivation of the Chinese validation cohort from The First Affiliated Hospital of Anhui Medical University (2015–2020), starting with 1,558 gastrointestinal cancer patients. Participants were excluded based on age, cancer diagnosis, and the availability of key data (sarcopenia and NPAR), resulting in two final analytical cohorts (NHANES: *n* = 1,586; Chinese: *n* = 705) for the comparative analysis.

The initial sample consisted of 80,630 participants from the NHANES 1999–2018 database. After excluding individuals aged < 20 years (*n* = 37,702), 42,928 participants remained. From this cohort, those without a cancer diagnosis were further excluded (*n* = 38,958), resulting in 3,970 cancer survivors. Subsequent exclusions due to missing data on sarcopenia (*n* = 2,223) and NPAR (*n* = 161) yielded a final analytical sample of 1,586 individuals from NHANES. Concurrently, an external validation cohort was derived from the First Affiliated Hospital of Anhui Medical University, comprising 1,558 gastrointestinal cancer patients treated between 2015 and 2020. Patients with gastrointestinal cancers were specifically selected for this validation cohort due to their high prevalence of cancer-related sarcopenia, which is often driven by nutritional impairment and systemic inflammation, making them a highly relevant population for evaluating an inflammatory-nutritional biomarker like NPAR (22). After excluding patients with missing sarcopenia (*n* = 693) and NPAR (*n* = 160) data, 705 patients were included in the validation analysis. The participant selection process for both cohorts is detailed in Figure 1.

### 2.3 Calculation of NPAR and other inflammatory indices

The NPAR was computed as: NPAR = (Neutrophil [%]/Albumin [g/dl]) × 10^2^. Where neutrophil percentage represents the proportion of neutrophils in total white blood cells, and albumin concentration is measured in grams per deciliter (23, 24). In the NHANES cohort, a complete blood count (CBC) with white blood cell differential, which includes the neutrophil percentage, was performed on automated hematology analyzers. Albumin concentration was measured using the standard biochemistry profile. Detailed laboratory procedures and instrument information are available on the NHANES website: https://wwwn.cdc.gov/nchs/nhanes/default.aspx. In the Chinese hospital-based cohort, the neutrophil percentage was determined from the CBC analysis, and serum albumin level was measured. Both assays were conducted using standard automated clinical analyzers in the hospital's central laboratory, following standardized clinical protocols. Concurrently, the following hematologic indices were calculated using absolute cell counts (expressed as × 10^9^/L) from CBC analysis: NLR = Neutrophil count (NC)/Lymphocyte count (LC); PLR = Platelet count (PC)/Lymphocyte count (LC); SII = Platelet count (PC) × Neutrophil count (NC)/Lymphocyte count (LC); SIRI = Neutrophil count (NC) × Monocyte count (MC)/Lymphocyte count (LC); AISI = Neutrophil count (NC) × Platelet count (PC) × Monocyte count (MC)/Lymphocyte count (LC).

### 2.4 Definition of sarcopenia

In this study, a widely accepted criterion that focuses on the assessment of skeletal muscle mass was used to define sarcopenia. Specifically, sarcopenia was identified as having a Skeletal Muscle Index (SMI) below a threshold value. For males, this threshold was set at 0.789, and for females, it was 0.512 (25, 26). This SMI criterion has been validated and widely used in recent studies of cancer survivors, supporting its applicability in our study context (27, 28). SMI was derived using appendicular skeletal muscle mass (ASM; kg) by BMI (kg/m^2^), where ASM denotes the aggregate muscle quantity in bilateral extremities measured by DXA. To ensure the robustness of our findings, we conducted sensitivity analyses using alternative diagnostic criteria. In the NHANES cohort, we applied the European Working Group on Sarcopenia in Older People (EWGSOP) consensus, defining sarcopenia as an appendicular skeletal muscle index (ASMI = ASM/height^2^) of < 7.26 kg/m^2^ for men and < 5.45 kg/m^2^ for women (29, 30), and the SARC-F questionnaire, with a score >4 indicating sarcopenia (31–33). In the Chinese hospital cohort, we applied the Asian Working Group for Sarcopenia (AWGS) 2019 consensus, defining low muscle mass as an ASMI < 7.0 kg/m^2^ for men and < 5.4 kg/m^2^ for women (5, 34).

### 2.5 Covariates

To mitigate confounding bias in assessing the NPAR-sarcopenia relationship, we implemented a hierarchical covariate adjustment framework across three tiers: demographic characteristics, lifestyle factors and health conditions, referring to published articles and clinical knowledge. In the NHANES cohort, demographic attributes covered age, gender, ethnicity, marital status, poverty index ratio (PIR), and educational level. Lifestyle elements included alcohol consumption and smoking habits. Smoking behavior was assessed through a detailed questionnaire, categorizing participants as never smoking, former smoking and now smoking. Drinking behavior was defined as never drinking, light drinking, moderate drinking and heavy drinking based on regular alcohol consumption patterns. Health conditions such as diabetes, hypertension, and hyperlipidemia were assessed based on physician diagnoses or self-reports. For the external validation cohort from the First Affiliated Hospital of Anhui Medical University, available covariates included age, gender, marital status, smoking (defined as never or ever smoking), alcohol consumption (defined as never or ever drinking), hypertension, diabetes, leukocyte count, and TNM stage, all of which were based on physician diagnoses or medical records. It should be noted that data on dietary protein intake were not available for the Chinese hospital-based cohort.

### 2.6 Statistical analysis

For both the NHANES cohort and the external validation cohort from the First Affiliated Hospital of Anhui Medical University, baseline characteristics of the study participants were summarized according to their sarcopenia status. Continuous variables were expressed as mean ± standard deviation, and categorical variables were presented as numbers and percentages. Group comparisons between participants with and without sarcopenia were conducted using Student's *t*-test for normally distributed continuous variables, the Mann-Whitney *U* test for non-normally distributed variables, and the Chi-square test (or Fisher's exact test where appropriate) for categorical variables.

The association between NPAR and sarcopenia was evaluated using multivariable logistic regression models, from which odds ratios (ORs) and their 95% confidence intervals (CIs) were derived. Three progressive adjustment strategies were employed: a crude model (unadjusted), an intermediate model adjusted for demographic covariates, and a fully adjusted model incorporating demographic, lifestyle, and clinical parameters. Continuous NPAR values were quartiled for trend testing.

To explore the potential nonlinear relationship between NPAR and sarcopenia, we employed generalized additive models (GAM) with smoothed curve fitting (penalized spline method). If a nonlinear association was identified, a two-piecewise linear regression model was further fitted to calculate the inflection point (threshold) using a likelihood ratio test.

The predictive performance of NPAR was quantified and compared with other novel inflammatory biomarkers (including NLR, PLR, SII, SIRI, and AISI) using receiver operating characteristic (ROC) curve analysis in both cohorts. The area under the curve (AUC) was calculated to assess the discriminative ability of each biomarker.

Additional analyses were conducted specifically in the NHANES cohort due to data availability. Mediation analyses were performed using a linear regression-based approach with bootstrapping (5,000 iterations) to examine the potential mediating roles of systemic inflammation (C-reactive protein, CRP) and dietary protein intake (DPI) in the association between NPAR and sarcopenia. The total, direct, and indirect effects were calculated, and the proportion mediated was reported.

Subgroup analyses based on the original sarcopenia diagnostic criteria and extensive sensitivity analyses employing multiple sarcopenia diagnostic criteria (including EWGSOP, AWGS 2019, and SARC-F) were conducted to rigorously evaluate the robustness of the primary findings. All statistical analyses were performed using EmpowerStats (v2.0) and R (v4.2.3), with the “pROC” package utilized for ROC analysis. A significance threshold of α = 0.05 was applied.

## 3 Results

### 3.1 Baseline characteristics

Baseline characteristics stratified by sarcopenia status are presented in Table 1 for the NHANES cohort (*n* = 1,586) and Table 2 for the hospital validation cohort (*n* = 705). In both cohorts, participants with sarcopenia were significantly older and had higher NPAR values compared to those without sarcopenia (all *p* < 0.001). While the NHANES cohort showed significant differences in ethnicity, education, lifestyle behaviors and metabolic comorbidities between groups, the hospital cohort of gastrointestinal cancer patients demonstrated no significant differences in sex distribution, marital status, diabetes prevalence, alcohol use, smoking status, TNM Stage, leukocyte or hypertension prevalence between sarcopenia and non-sarcopenia groups (all *p* > 0.05). Furthermore, the distribution of NPAR across key demographic and clinical subgroups is detailed in Tables 1, 2. For instance, in the NHANES cohort, higher NPAR levels were observed in participants with conditions such as diabetes (Yes: 14.70 ± 2.87) compared to those without (No: 14.04 ± 2.66). Similar trends were noted across other subgroups, providing a comprehensive overview of the biomarker's variation within the study populations.

**Table 1:** Baseline study population characteristics (weighted).

**Characteristic**	**Total**	**Without sarcopenia**	**Sarcopenia**	***P*-value**	**NPAR (mean ±SD)**
Sample size (example)	1,586	1,318	168	1	
Age (year), mean ± SD	56.21 ± 0.46	55.01 ± 0.47	64.86 ± 1.34	< 0.0001	
NPAR, mean ± SD	14.06 ± 0.09	13.93 ± 0.10	14.96 ± 0.21	< 0.0001	
**Sex**, ***n*** **(%)**				0.0118	
Female	61.51 (58.72, 64.23)	62.87 (59.79, 65.85)	51.75 (43.39, 60.01)		14.26 ± 2.64
Male	38.49 (35.77, 41.28)	37.13 (34.15, 40.21)	48.25 (39.99, 56.61)		14.15 ± 2.84
**Race**, ***n*** **(%)**				< 0.0001	
Mexican American	2.49 (1.86, 3.34)	2.04 (1.47, 2.82)	5.77 (3.68, 8.94)		14.13 ± 2.49
Other Hispanic	2.81 (1.76, 4.47)	2.27 (1.49, 3.44)	6.75 (2.72, 15.75)		13.65 ± 2.10
Non-Hispanic white	86.57 (84.21, 88.63)	87.14 (84.84, 89.13)	82.52 (74.27, 88.53)		14.30 ± 2.71
Non-Hispanic black	4.99 (4.02, 6.17)	5.44 (4.39, 6.74)	1.69 (0.83, 3.42)		13.84 ± 3.20
Other race	3.13 (2.26, 4.33)	3.12 (2.20, 4.40)	3.27 (1.48, 7.05)		14.40 ± 2.45
**Education**, ***n*** **(%)**				< 0.0001	
< High school	15.19 (12.98, 17.70)	13.72 (11.49, 16.30)	25.83 (19.67, 33.13)		14.25 ± 2.92
High school	23.42 (20.69, 26.39)	22.69 (19.78, 25.90)	28.66 (21.91, 36.53)		14.03 ± 2.61
>High school	61.39 (58.02, 64.64)	63.59 (60.04, 67.00)	45.50 (38.14, 53.07)		14.27 ± 2.70
**Marital status**, ***n*** **(%)**				0.2505	
Married/living with a partner	67.96 (65.06, 70.74)	68.21 (65.15, 71.12)	66.20 (58.44, 73.17)		14.27 ± 2.70
Divorced/separated/widowed	25.35 (22.78, 28.10)	24.83 (22.16, 27.71)	29.05 (22.58, 36.51)		14.67 ± 2.98
Never married	6.69 (5.31, 8.40)	6.96 (5.43, 8.88)	4.75 (2.83, 7.87)		14.34 ± 3.03
**PIR**, ***n*** **(%)**				0.9936	
< 1	10.52 (8.39, 13.10)	10.51 (8.48, 12.97)	10.54 (5.80, 18.39)		14.41 ± 2.79
≥1	89.48 (86.90, 91.61)	89.49 (87.03, 91.52)	89.46 (81.61, 94.20)		14.18 ± 2.72
**Diabetes mellitus**, ***n*** **(%)**				0.0001	
No	78.67 (75.79, 81.30)	80.63 (77.61, 83.33)	64.53 (55.20, 72.88)		14.04 ± 2.66
Yes	21.33 (18.70, 24.21)	19.37 (16.67, 22.39)	35.47 (27.12, 44.80)		14.70 ± 2.87
**Alcohol user**, ***n*** **(%)**				0.0021	
Never drinking	10.18 (8.57, 12.05)	9.68 (7.92, 11.77)	13.77 (9.23, 20.05)		14.47 ± 2.88
Light drinking	57.06 (53.58, 60.48)	55.43 (51.47, 59.33)	68.82 (59.64, 76.74)		14.24 ± 2.80
Moderate drinking	16.39 (14.22, 18.83)	17.26 (14.88, 19.92)	10.17 (5.37, 18.40)		13.95 ± 2.31
Heavy drinking	16.37 (14.06, 18.96)	17.63 (15.03, 20.58)	7.24 (3.83, 13.27)		14.04 ± 2.58
**Smoke**, ***n*** **(%)**				0.0014	
Never	41.78 (38.79, 44.83)	41.85 (38.85, 44.92)	41.25 (33.84, 49.08)		14.29 ± 2.57
Former	36.47 (33.55, 39.50)	35.09 (32.16, 38.15)	46.42 (39.25, 53.74)		14.10 ± 2.92
Now	21.75 (19.28, 24.43)	23.05 (20.47, 25.85)	12.33 (7.51, 19.59)		14.27 ± 2.67
**Hypertension**, ***n*** **(%)**				< 0.0001	
No	49.42 (45.74, 53.10)	52.26 (48.52, 55.97)	28.92 (21.90, 37.11)		13.97 ± 2.69
Yes	50.58 (46.90, 54.26)	47.74 (44.03, 51.48)	71.08 (62.89, 78.10)		14.38 ± 2.75
**Hyperlipidemia (%)**				< 0.0001	
No	22.73 (20.12, 25.58)	24.21 (21.38, 27.27)	12.12 (8.53, 16.94)		14.33 ± 2.81
Yes	77.27 (74.42, 79.88)	75.79 (72.73, 78.62)	87.88 (83.06, 91.47)		14.18 ± 2.71

**Table 2:** Baseline study population characteristics in the hospital cohort.

**Characteristic**	**Total**	**Without sarcopenia**	**Sarcopenia**	***P*-value**	**NPAR (mean ±SD)**
Sample size (example)	705	543	162	1	
Age (year), mean ± SD	62.23 ± 10.15	61.27 ± 10.30	65.45 ± 8.94	< 0.001	
NPAR, mean ± SD	14.08 ± 3.34	14.01 ± 3.48	14.96 ± 3.29	< 0.001	
Leukocyte (10^9^/L), mean ± SD	5.58 ± 1.94	5.57 ± 2.04	5.63 ± 1.57	0.203	
**Sex**, ***n*** **(%)**				0.986	
Female	165 (23.40%)	127 (23.39)	38 (23.46)		13.48 ± 3.65
Male	540 (76.60%)	416 (76.61)	124 (76.54)		13.66 ± 3.35
**Marital status**, ***n*** **(%)**				0.906	
Married/living with a partner	568 (80.57%)	438 (80.67)	130 (80.25)		13.36 ± 3.20
Never married/divorced/separated/widowed	137 (19.43%)	105 (19.33)	32 (19.75)		13.68 ± 3.47
**Diabetes mellitus**, ***n*** **(%)**				0.409	
No	614 (87.09)	476 (87.66)	138 (85.19)		13.65 ± 3.38
Yes	91 (12.91)	67 (12.34)	24 (14.81)		13.78 ± 3.75
**Alcohol user**, ***n*** **(%)**				0.308	
Never drinking	591 (83.82)	451 (83.06)	140 (86.42)		13.63 ± 3.55
Drinking	114 (16.18)	92 (16.94)	22 (13.58)		13.58 ± 3.09
**Smoke**, ***n*** **(%)**				0.203	
Never smoking	558 (79.14)	424 (78.08)	134 (82.71)		13.63 ± 3.56
Smoking	147 (20.86)	119 (21.92)	28 (17.29)		13.47 ± 2.92
**Hypertension**, ***n*** **(%)**				0.972	
No	536 (76.02)	413 (76.06)	123 (75.92)		13.72 ± 3.59
Yes	169 (23.98)	130 (23.94)	39 (24.08)		13.37 ± 2.84
**TNM_Stage**				0.856	
I	113 (16.03%)	89 (16.39)	24 (14.81)		13.71 ± 3.13
II	151 (21.42%)	116 (21.36)	35 (21.60)		13.53 ± 3.35
III	304 (43.12%)	236 (43.46)	68 (41.98)		13.61 ± 3.63
IV	137 (19.43%)	102 (18.78)	35 (21.60)		13.66 ± 3.30

### 3.2 Consistent association of NPAR with sarcopenia across cohorts with dose-response relationship

A consistent positive association between NPAR and sarcopenia was observed in both the NHANES and hospital-based validation cohorts across multiple adjusted models (Tables 3, 4). In the hospital cohort, per-unit increase in NPAR as a continuous variable was significantly associated with elevated sarcopenia risk in the unadjusted model (OR = 1.09, 95% CI: 1.03–1.14, *p* = 0.0010), after adjusting for age, sex, and marital status (OR = 1.09, 95% CI: 1.04–1.15, *p* = 0.0005), and further controlling for smoking, alcohol use, hypertension, diabetes, leukocyte count, and TNM stage (OR = 1.10, 95% CI: 1.04–1.14, *p* = 0.0007). When analyzed in quartiles, participants in the highest NPAR quartile (Q4) had markedly increased odds of sarcopenia compared to the lowest quartile (Q1), with ORs of 2.80 (95% CI: 1.65–4.74, *p* < 0.0001), 3.03 (95% CI: 1.77–5.20, *p* < 0.0001), and 2.99 (95% CI: 1.63–5.32, *p* = 0.0003) in Models 1, 2, and 3, respectively. A significant dose-response trend was consistently identified (*p* for trend: 0.0003, < 0.0001, and 0.0003 in Models 1, 2, and 3).

**Table 3:** The association between NPAR and sarcopenia in individuals with cancer (weighted).

**Variable**	**Model 1 OR (95% CI) *P*-value**	**Model 2 OR (95% CI) *P*-value**	**Model 3 OR (95% CI) *P*-value**
NPAR (continuousa)	1.17 (1.09, 1.25) < 0.0001	1.13 (1.05, 1.23) 0.0029	1.14 (1.06, 1.22) 0.0007
**NPAR (quartile)**			
Q1	[Reference]	[Reference]	[Reference]
Q2	0.88 (0.51, 1.53) 0.6593	0.90 (0.51, 1.58) 0.7048	0.92 (0.52, 1.62) 0.7647
Q3	1.11 (0.66, 1.88) 0.6978	1.00 (0.57, 1.76) 0.9926	1.06 (0.61, 1.84) 0.8494
Q4	2.17 (1.36, 3.47) 0.0017	1.78 (1.05, 3.00) 0.0347	1.79 (1.05, 3.06) 0.0349
*P* for trend	0.0017	0.0354	0.0304

**Table 4:** The association between NPAR and sarcopenia in individuals with cancer in hospital cohort.

**Variable**	**Model 1 OR (95% CI) *P*-value**	**Model 2 OR (95% CI) *P*-value**	**Model 3 OR (95% CI) *P*-value**
NPAR (continuousa)	1.09 (1.03, 1.14) 0.0010	1.09 (1.04, 1.15) 0.0005	1.10 (1.04, 1.14) 0.0007
**NPAR (quartile)**			
Q1	[Reference]	[Reference]	[Reference]
Q2	1.83 (1.06, 3.18) 0.0303	1.84 (1.05, 3.22) 0.0319	1.77 (0.96, 3.23) 0.065
Q3	1.78 (1.02, 3.08) 0.0409	1.91 (1.09, 3.34) 0.0243	1.84 (0.98, 3.38) 0.0536
Q4	2.80 (1.65, 4.74) 0.0001	3.03 (1.77, 5.20) < 0.0001	2.99 (1.63, 5.32) 0.0003
*P* for trend	0.0003	< 0.0001	0.0003

### 3.3 Smooth curve fitting and threshold effect analysis

**Figure 2:**
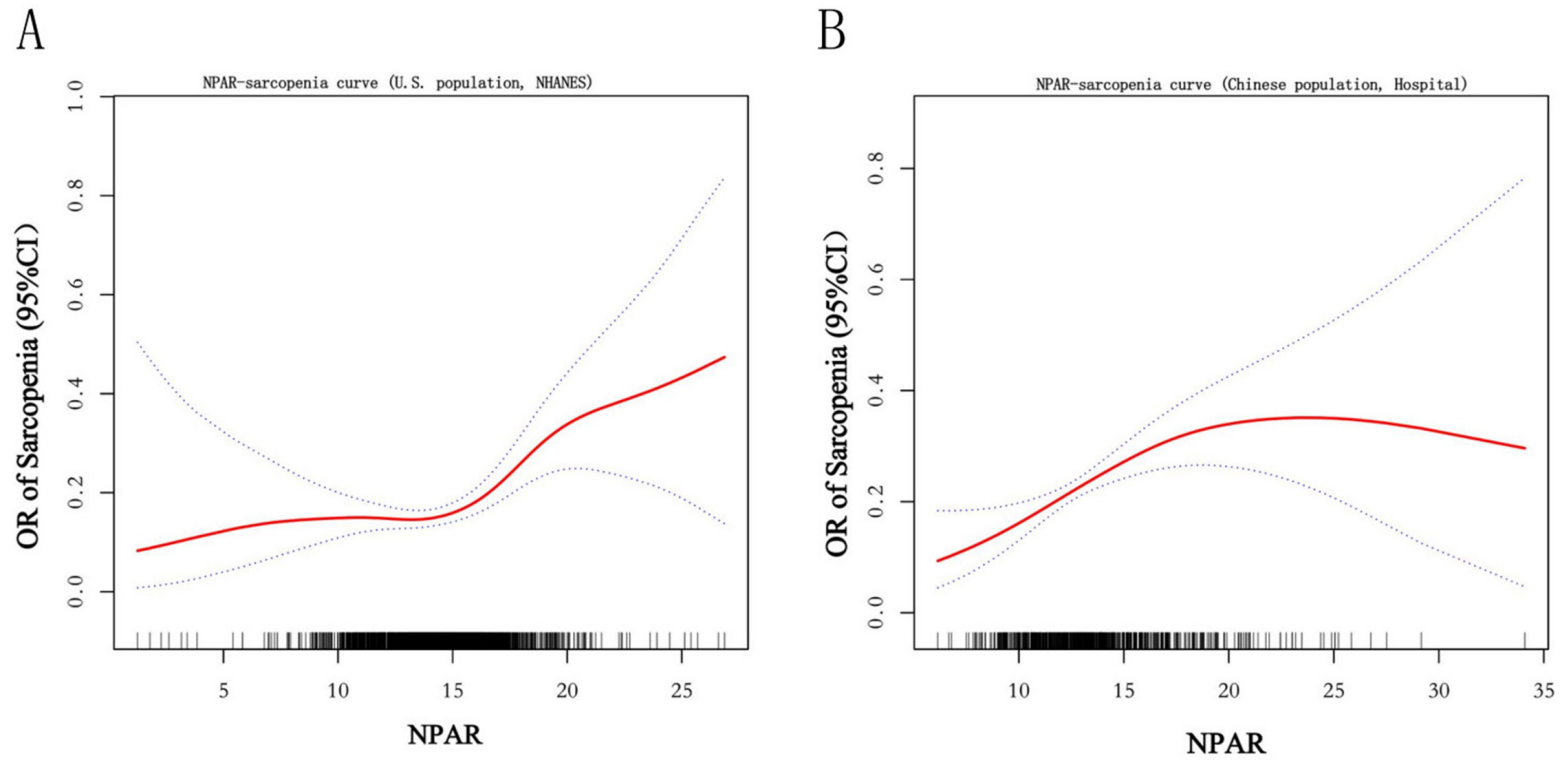
Smooth curve. The figure depicts the unadjusted, nonlinear relationship between the Neutrophil Percentage-to-Albumin Ratio (NPAR) and the odds of sarcopenia using generalized additive models. The solid curve represents the estimated odds ratio (OR), and the shaded area represents the 95% confidence interval. Results are presented for the **(A)** U.S. cohort (NHANES) and the **(B)** Chinese hospital-based validation cohort.

The relationship between NPAR and sarcopenia was further explored using smooth curve fitting analysis (Figure 2; unadjusted for covariates in Model 1). The results revealed a complex association that encompassed both linear and nonlinear components. Threshold effect analysis (Tables 5, 6) was conducted to identify critical points in this relationship. In the NHANES cohort, a threshold was identified at NPAR = 14.7, below which the association was non-significant (OR = 1.00, 95% CI: 0.91–1.10, *p* = 0.976) and above which each unit increase in NPAR significantly raised sarcopenia risk by 21% (OR = 1.21, 95% CI: 1.11–1.33, *p* < 0.001). Conversely, the hospital cohort exhibited a distinct threshold at NPAR = 17.07, with a significant effect below this value (OR = 1.17, 95% CI: 1.08–1.27, *p* = 0.0002) but no significant association above it (OR = 0.96, 95% CI: 0.85–1.09, *p* = 0.5319). Notably, both cohorts demonstrated statistically significant nonlinearity (likelihood ratio test *p* = 0.016 and *p* = 0.022, respectively).

**Table 5:** Threshold effect analysis in NHANES cohort.

**Scenario**	**Analysis type**	**Indicator**	**OR (95% CI)**	***P*-value**
Scenario I	Linear effect	Overall effect	1.11 (1.05, 1.17)	< 0.001
Scenario II	Non-linear effect	Threshold (K)	14.7	–
Scenario II	Non-linear effect	Effect below K	1.00 (0.91, 1.10)	0.976
Scenario II	Non-linear effect	Effect above K	1.21 (1.11, 1.33)	< 0.001
Scenario II	Non-linear effect	Difference in effects (2 vs. 1)	1.21 (1.04, 1.41)	0.014
Scenario II	Non-linear effect	Likelihood ratio test	–	0.016

**Table 6:** Threshold effect analysis in hospital cohort.

**Scenario**	**Analysis type**	**Indicator**	**OR (95% CI)**	***P*-value**
Scenario I	Linear effect	Overall effect	1.09 (1.03, 1.14)	0.001
Scenario II	Non-linear effect	Threshold (K)	17.07	–
Scenario II	Non-linear effect	Effect below K	1.17 (1.08, 1.27)	0.0002
Scenario II	Non-linear effect	Effect above K	0.96 (0.85, 1.09)	0.5319
Scenario II	Non-linear effect	Difference in effects (2 vs. 1)	0.82 (0.69, 0.98)	0.0270
Scenario II	Non-linear effect	Likelihood ratio test	–	0.022

### 3.4 ROC curve analysis

**Figure 3:**
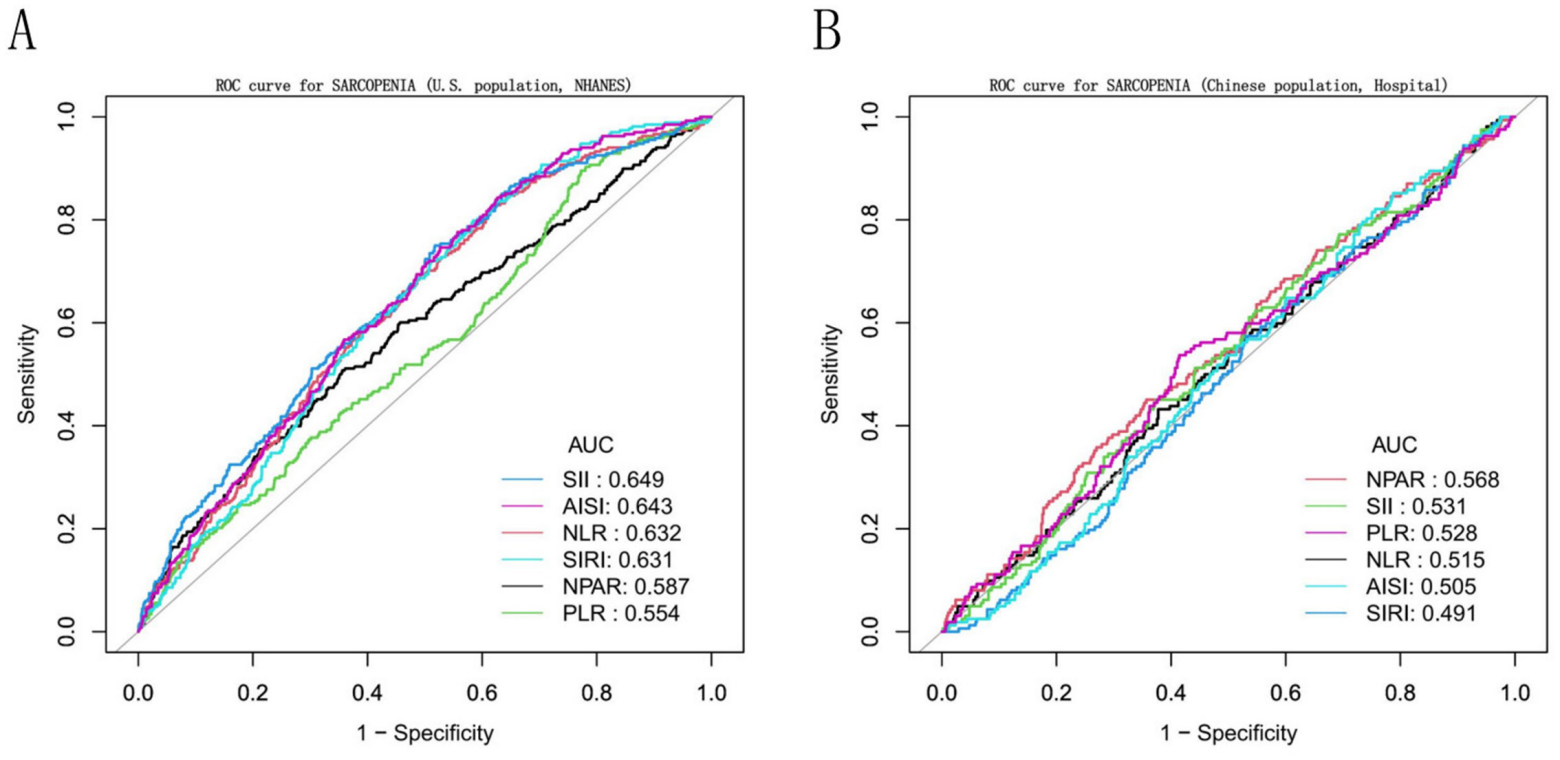
Receiver operating characteristic (ROC) curve between NPAR and other inflammatory biomarkers for sarcopenia in cancer survivors. The figure displays ROC curves evaluating the discriminative ability of the Neutrophil Percentage-to-Albumin Ratio (NPAR) and other systemic inflammatory indices (NLR, PLR, SII, SIRI, AISI) for sarcopenia in the **(A)** U.S. NHANES cohort and the **(B)** Chinese hospital-based validation cohort. The area under the curve (AUC) value for each biomarker is provided in the legend.

The ROC analysis was performed to evaluate the predictive performance of NPAR and other inflammatory biomarkers for sarcopenia in both cohorts (Figure 3). In the NHANES cohort, the SII demonstrated the highest discriminative ability (AUC = 0.649), followed by AISI (0.643), NLR (0.632), SIRI (0.631), NPAR (0.587), and PLR (0.554). In contrast, within the hospital validation cohort, NPAR achieved an AUC of 0.568, outperforming other biomarkers including PLR (0.528), SII (0.531), NLR (0.515), AISI (0.505), and SIRI (0.491).

### 3.5 Subgroup analysis

**Figure 4:**
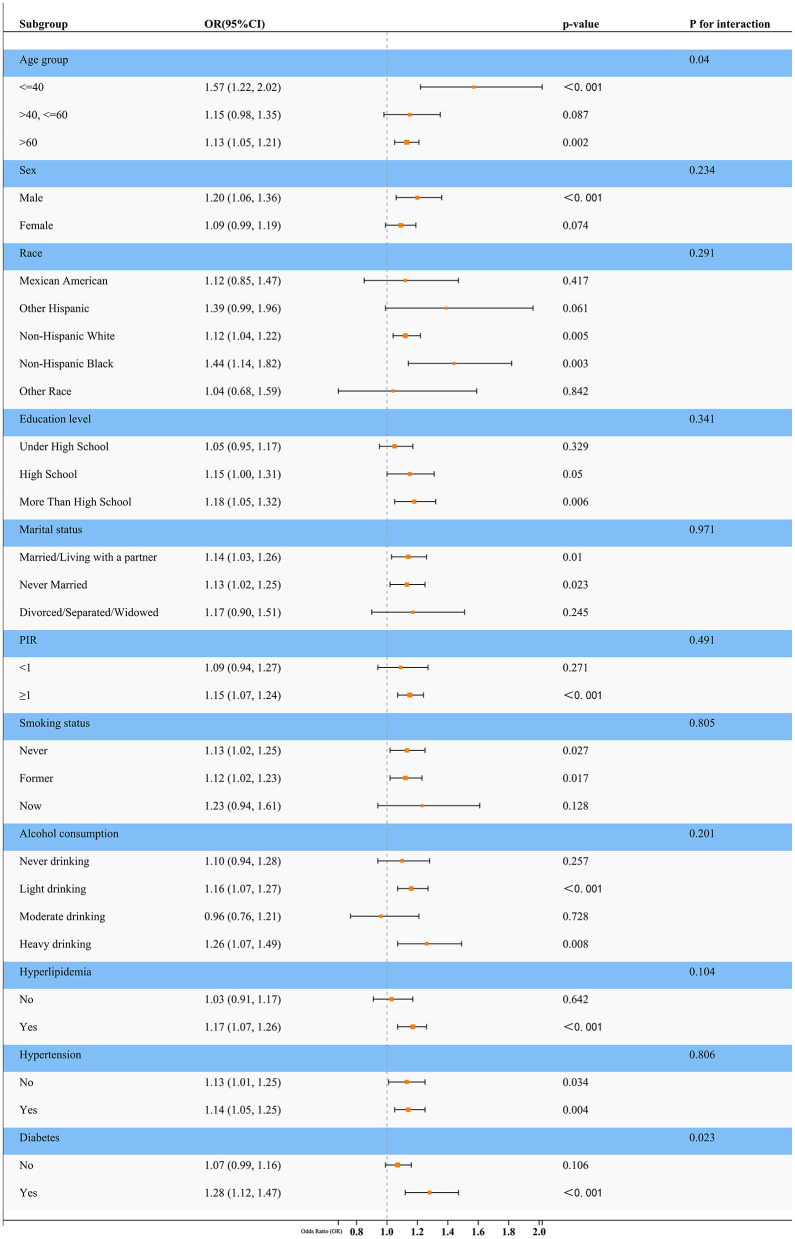
Forest plot for subgroup analysis. This forest plot presents the odds ratios (ORs) and 95% confidence intervals (CIs) for the association between NPAR (per unit increase) and sarcopenia across various demographic and clinical subgroups in the NHANES cohort. A *p*-value for interaction was calculated for each subgroup to assess effect modification. The analysis revealed significant interactions for age (*p* for interaction = 0.04) and diabetes status (*p* for interaction = 0.023), indicating that the strength of the NPAR-sarcopenia association varies within these subgroups. No significant interactions were observed for the other covariates, suggesting a consistent association across those strata.

In NHANES cohort, subgroup analyses (Figure 4) were performed to assess potential interactions between covariates. Significant interactions were identified in the age group (*p* for interaction = 0.04) and diabetes status (*p* for interaction = 0.023). Specifically, the association between NPAR and sarcopenia varied across age strata: the strongest effect was observed in individuals aged < 40 years (OR = 1.57, 95% CI: 1.22–2.02, *p* < 0.001), with attenuated effects in older subgroups (e.g., >60 years: OR = 1.13, 95% CI: 1.05–1.21, *p* = 0.002). Similarly, the relationship was more pronounced in participants with diabetes (OR = 1.28, 95% CI: 1.12–1.47, *p* < 0.001) compared to those without (OR = 1.07, 95% CI: 0.99–1.16, *p* = 0.106). No significant interactions were detected for sex, race, education level, marital status, PIR, smoking status, alcohol consumption, hyperlipidemia, or hypertension (all *p* for interaction >0.05). These findings suggest that age and diabetes status may modify the impact of NPAR on sarcopenia risk, whereas other demographic and clinical factors do not exhibit substantial effect heterogeneity.

### 3.6 Mediation analyses of inflammatory and nutritional pathways

**Figure 5:**
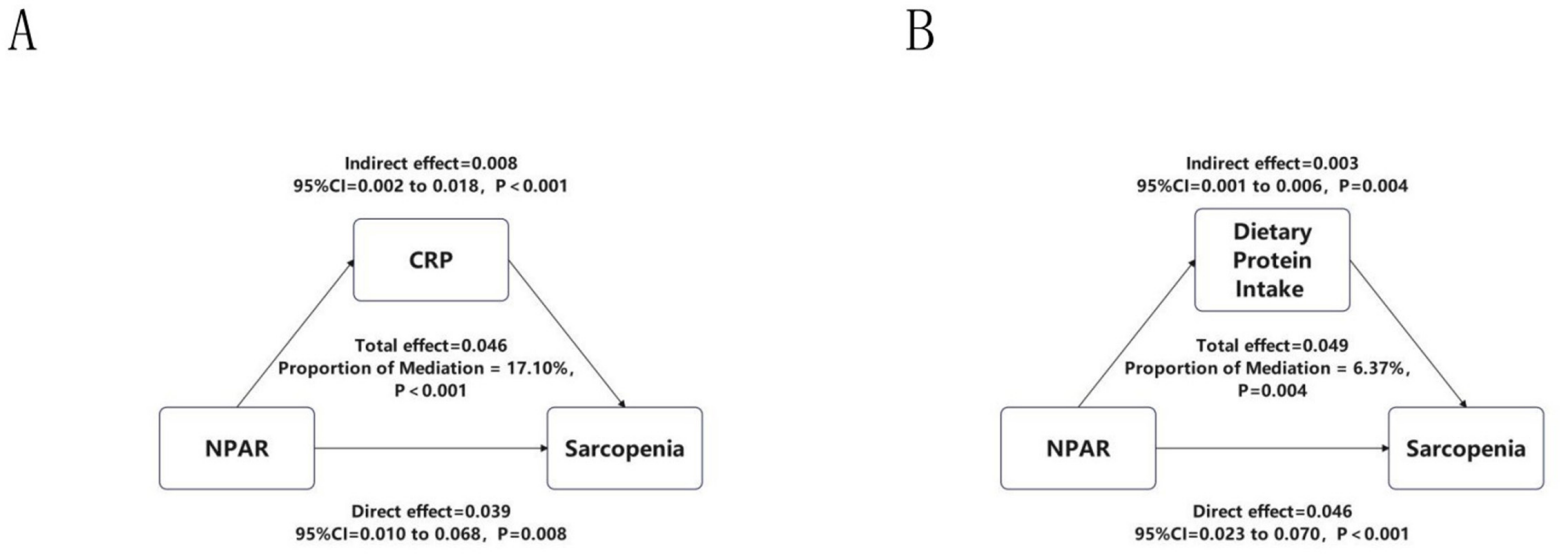
Mediation analyses of CRP and DPI. This figure illustrates the mediating effects of **(A)** systemic inflammation, assessed by C-reactive protein (CRP), and **(B)** nutritional status, assessed by dietary protein intake (DPI), on the relationship between NPAR and sarcopenia. This analysis was performed specifically in the NHANES cohort. The total effect, direct effect, and indirect (mediating) effect are presented as beta coefficients with 95% confidence intervals (calculated using the bootstrap method with 5,000 iterations) and *p*-values. The proportion of the total effect mediated by each pathway is indicated.

To further explore mechanistic pathways underlying the association between NPAR and sarcopenia, as shown in Figure 5, we evaluated two biologically plausible mediators: systemic inflammation (CRP) and nutritional adequacy (DPI). It is important to note that this mediation analysis was conducted exclusively in the NHANES cohort due to data availability. Mediation analyses revealed a significant indirect effect of CRP (β = 0.008, 95% CI: 0.002–0.018, *p* < 0.001), accounting for 17.10% of the total effect (β = 0.046, *p* < 0.001), indicating a substantial mediation by inflammatory pathways. In parallel, DPI exhibited a smaller yet statistically robust mediation effect (β = 0.003, 95% CI: 0.001–0.006, *p* = 0.004), explaining 6.37% of the total effect (β = 0.049, *p* = 0.004), consistent with evidence linking suboptimal protein intake to muscle atrophy.

### 3.7 Sensitivity analyses

**Figure 6:**
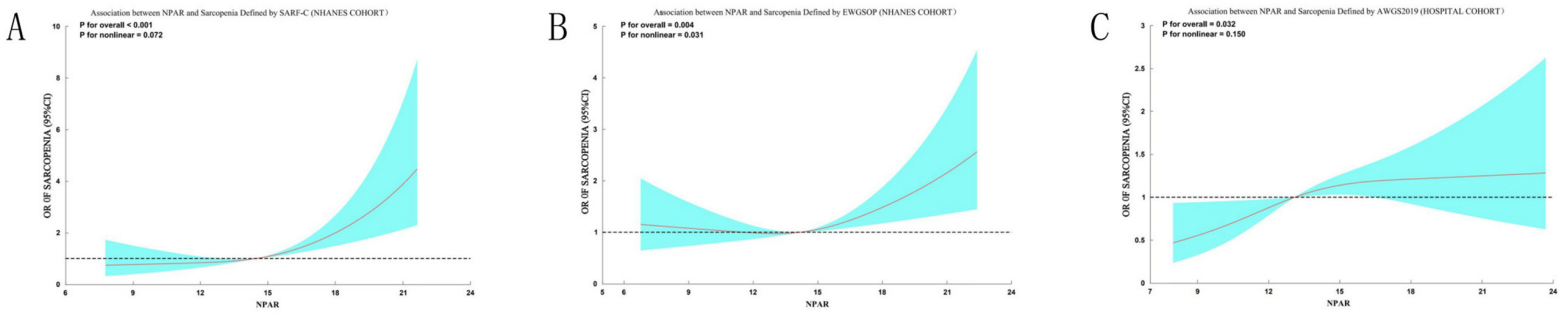
Sensitivity analyses of the association between NPAR and sarcopenia using alternative diagnostic criteria. Restricted cubic spline analyses demonstrate the consistent positive association between NPAR and sarcopenia risk when defined by different criteria: **(A)** SARC-F questionnaire (score > 4) in the NHANES cohort; **(B)** EWGSOP criteria (ASMI < 7.26 kg/m^2^ for men, < 5.45 kg/m^2^ for women) in the NHANES cohort; **(C)** AWGS 2019 criteria (ASMI < 7.0 kg/m^2^ for men, < 5.4 kg/m^2^ for women) in the hospital cohort. Solid curves represent odds ratios (ORs), and shaded areas indicate 95% confidence intervals. All models used three knots and were unadjusted for covariates. The significant overall associations (*p* for overall < 0.05 for all) and detailed threshold effect analyses (Supplementary Tables S1–S3) confirm the robustness of the NPAR-sarcopenia relationship across multiple diagnostic frameworks.

To rigorously evaluate the robustness of our primary findings against variations in sarcopenia definition, we conducted comprehensive sensitivity analyses using three alternative diagnostic criteria. When sarcopenia was redefined using the SARC-F questionnaire in the NHANES cohort, restricted cubic spline (RCS) analysis confirmed a significant positive association with NPAR (*p* for overall < 0.001). Threshold effect analysis identified an inflection point at NPAR = 14.9, below which the association was not significant (OR = 1.04, 95% CI: 0.92–1.17, *p* = 0.521) and above which each unit increase in NPAR significantly elevated sarcopenia risk by 26% (OR = 1.26, 95% CI: 1.14–1.40, *p* < 0.001; Supplementary Table S1). Similarly, application of the EWGSOP criteria in the NHANES cohort also revealed a significant overall association (*p* for overall = 0.0146) and a nonlinear relationship (*p* for nonlinear = 0.031). The risk of EWGSOP-defined sarcopenia increased significantly once NPAR exceeded a threshold of 13.2 (OR = 1.11, 95% CI: 1.05–1.18, *p* < 0.001; Supplementary Table S2). In the Chinese hospital cohort, using the AWGS 2019 criteria, a significant linear association was observed (OR = 1.06, 95% CI: 1.01–1.11, *p* = 0.021). The RCS curves for all three alternative criteria are presented in Figure 6.

## 4 Discussion

The investigation identifies elevated NPAR as an independent predictor of sarcopenia risk in both American and Chinese oncologic cohorts, demonstrating consistent dose-response relationships across diverse populations. This association remained robust after comprehensive adjustment for demographic, lifestyle, and clinical confounders in both the NHANES and hospital-based validation cohorts. Notably, threshold analysis revealed that sarcopenia risk increased with rising NPAR within specific critical ranges—when NPAR exceeded 14.7 in the NHANES cohort and remained below 17.07 in the hospital cohort—highlighting a nonlinear yet consistently positive association within these intervals. While systemic inflammatory indices, including SII, AISI, and NLR, exhibited marginally higher diagnostic accuracy in the NHANES cohort, NPAR demonstrated superior performance compared to other biomarkers in the clinical validation cohort, underscoring its particular utility in a patient care setting.

The consistent association of NPAR with sarcopenia underscores its utility as a composite biomarker. A key strength of NPAR lies in its ability to integrate two fundamental pathophysiological drivers of cancer-related sarcopenia: neutrophil-derived inflammatory activity and hypoalbuminemia-associated nutritional deficiency (35–37). A pertinent consideration is that an identical NPAR value can result from differing contributions of its components. However, we posit that this integrated nature is precisely its strength for assessing a multifactorial syndrome. It simultaneously quantifies the inflammatory drive for muscle catabolism (via neutrophil percentage) and the nutritional capacity for muscle anabolism (via albumin). Our mediation analyses provide empirical support for this dual-pathway model, demonstrating that both systemic inflammation (CRP) and nutritional intake (dietary protein) significantly mediated the NPAR-sarcopenia relationship (Figure 5). This confirms that the biological pathways represented by both constituents of NPAR are mechanistically involved in muscle wasting.

The observed association may be mechanistically linked to the interplay between neutrophilia and hypoalbuminemia. Neutrophils, key mediators of cancer-related inflammation, release reactive oxygen species and proteolytic enzymes that promote muscle protein degradation (38). Concurrently, hypoalbuminemia—a marker of malnutrition and chronic disease—compromises muscle synthesis and repair, further accelerating sarcopenia progression (39). Our subgroup analyses revealed effect modification by age and diabetes status, suggesting that older patients and those with metabolic dysregulation may be more vulnerable to NPAR-driven sarcopenia. This aligns with studies emphasizing the role of age-related immunosenescence and insulin resistance in exacerbating inflammatory muscle loss (40, 41).

Clinically, the primary utility of NPAR lies in risk stratification and initial screening during routine oncology care. Its accessibility through routine blood tests positions it as a pragmatic tool, serving not as a standalone diagnostic, but as an objective “red flag.” This addresses a key practical need by complementing more resource-intensive or subjective assessments. An elevated NPAR should prompt definitive assessments (e.g., DXA scans) and guide timely, targeted interventions. For instance, a ratio driven predominantly by neutrophilia might prioritize anti-inflammatory strategies, whereas one driven by hypoalbuminemia would necessitate aggressive nutritional support. This actionable nature, stemming from its ability to integrate two critical pathological states, is a key clinical advantage. While this study focused on cancer patients, NPAR's predictive utility across diverse conditions (15, 17, 42) reinforces its versatility as a biomarker of the inflammation-nutrition axis, a common pathway in many chronic diseases.

Several limitations should be considered when interpreting our findings. First, the use of cross-sectional data in each cohort precludes the establishment of causal relationships between NPAR and sarcopenia; longitudinal or interventional studies are required to confirm temporal associations and its predictive utility over time. Second, in the hospital cohort, NPAR was obtained preoperatively, and its levels may be influenced by underlying cancer or pre-treatment conditions, which could bias the observed associations. Future studies incorporating serial body composition assessments from pre-diagnosis through treatment are needed to establish the dynamic prognostic value of NPAR. Third, the generalizability of our findings from the clinical cohort to other cancer populations may be limited, as it consisted exclusively of patients with gastrointestinal cancers, who were selected due to the high prevalence of sarcopenia driven by nutritional issues and inflammation in this group. Fourth, certain limitations in data availability should be noted, despite expanding adjustments in the hospital cohort to include TNM stage and leukocyte count, other key oncologic variables (e.g., treatment modality, active infection) were not available. Furthermore, dietary protein intake was not available in this cohort, which precluded external validation of the nutritional mediation analysis and may influence the comparability of effect estimates between the two study populations. Future research should also compare NPAR's performance against sarcopenia-specific biomarkers (e.g., myostatin, IGF-1) (43) and validate its prognostic value in large, multicenter prospective cohorts.

## 5 Conclusions

This study establishes NPAR as a practical and informative biomarker for sarcopenia risk stratification in cancer patients, integrating both inflammatory and nutritional dimensions into a single accessible measure. Its derivation from routine blood tests enables cost-effective early identification of high-risk individuals during standard oncology care. These findings support the use of NPAR in guiding timely interventions—such as nutritional therapy and anti-inflammatory support—to preserve muscle mass and improve clinical outcomes in cancer populations.

## Data Availability

The raw data supporting the conclusions of this article will be made available by the authors, without undue reservation.

## References

[1] Prado CM Lieffers JR McCargar LJ Reiman T Sawyer MB Martin L . Prevalence and clinical implications of sarcopenic obesity in patients with solid tumours of the respiratory and gastrointestinal tracts: a population-based study. Lancet Oncol. (2008) 9:629–35. doi: 10.1016/S1470-2045(08)70153-0 18539529

[2] Baracos VE Arribas L. Sarcopenic obesity: hidden muscle wasting and its impact for survival and complications of cancer therapy. Ann Oncol. (2018) 29(suppl_2):ii1–9. doi: 10.1093/annonc/mdx810 32169202

[3] Prado CM Cushen SJ Orsso CE Ryan AM. Sarcopenia and cachexia in the era of obesity: clinical and nutritional impact. Proc Nutr Soc. (2016) 75:188–98. doi: 10.1017/S0029665115004279 26743210

[4] Biolo G Cederholm T Muscaritoli M. Muscle contractile and metabolic dysfunction is a common feature of sarcopenia of aging and chronic diseases: from sarcopenic obesity to cachexia. Clin Nutr. (2014) 33:737–48. doi: 10.1016/j.clnu.2014.03.007 24785098

[5] Chen LK Woo J Assantachai P Auyeung TW Chou MY Iijima K . Asian working group for sarcopenia: 2019 consensus update on sarcopenia diagnosis and treatment. J Am Med Dir Assoc. (2020) 21:300–7.e2. doi: 10.1016/j.jamda.2019.12.012 32033882

[6] Cruz-Jentoft AJ Bahat G Bauer J Boirie Y Bruyère O Cederholm T . Sarcopenia: revised European consensus on definition and diagnosis. Age Ageing. (2019) 48:16–31. doi: 10.1093/ageing/afy169 30312372 PMC6322506

[7] Lipshitz M Visser J Anderson R Nel DG Smit T Steel HC . Emerging markers of cancer cachexia and their relationship to sarcopenia. J Cancer Res Clin Oncol. (2023) 149:17511–27. doi: 10.1007/s00432-023-05465-9 37906352 PMC10657295

[8] Setiawan T Sari IN Wijaya YT Julianto NM Muhammad JA Lee H . Cancer cachexia: molecular mechanisms and treatment strategies. J Hematol Oncol. (2023) 16:54. doi: 10.1186/s13045-023-01454-0 37217930 PMC10204324

[9] Compton SLE Heymsfield SB Brown JC. Nutritional mechanisms of cancer cachexia. Annu Rev Nutr. (2024) 44:77–98. doi: 10.1146/annurev-nutr-062122-015646 39207878

[10] Xie H Jia P Wei L Ruan G Zhang H Ge Y . Evaluation and validation of neutrophil to albumin ratio as a promising prognostic marker for all-cause mortality in patients with cancer: a multicenter cohort study. Nutrition. (2024) 121:112365. doi: 10.1016/j.nut.2024.112365 38377700

[11] Lv XN Shen YQ Li ZQ Deng L Wang ZJ Cheng J . Neutrophil percentage to albumin ratio is associated with stroke-associated pneumonia and poor outcome in patients with spontaneous intracerebral hemorrhage. Front Immunol. (2023) 14:1173718. doi: 10.3389/fimmu.2023.1173718 37388726 PMC10300413

[12] Zhou Z Zhang Y Pan Y Yang X Li L Gao C . A novel neutrophil-based biomarker to monitor disease activity and predict response to infliximab therapy in patients with ulcerative colitis. Front Med. (2022) 9:872831. doi: 10.3389/fmed.2022.872831 35572985 PMC9092064

[13] He X Dai F Zhang X Pan J. The neutrophil percentage-to-albumin ratio is related to the occurrence of diabetic retinopathy. J Clin Lab Anal. (2022) 36:e24334. doi: 10.1002/jcla.24334 35285099 PMC8993596

[14] Shen G Liu Y Zhou C Luo W Yang YX Guo S . Associations between neutrophil-percentage-to-albumin ratio level and all-cause mortality and cardiovascular disease-cause mortality in diabetes population. BMC Public Health. (2025) 25:401. doi: 10.1186/s12889-024-20924-9 39891098 PMC11786390

[15] Wang Y Shang X Zhang Y Zhang Y Shen W Wu Q . The association between neutrophil to high-density lipoprotein cholesterol ratio and gallstones: a cross-sectional study. BMC Public Health. (2025) 25:157. doi: 10.1186/s12889-025-21392-5 39810139 PMC11734447

[16] Jimenez-Gutierrez GE Martínez-Gómez LE Martínez-Armenta C Pineda C Martínez-Nava GA Lopez-Reyes A. Molecular mechanisms of inflammation in sarcopenia: diagnosis and therapeutic update. Cells. (2022) 11:2359. doi: 10.3390/cells11152359 35954203 PMC9367570

[17] Ding W La R Wang S He Z Jiang D Zhang Z . Associations between neutrophil percentage to albumin ratio and rheumatoid arthritis versus osteoarthritis: a comprehensive analysis utilizing the NHANES database. Front Immunol. (2025) 16:1436311. doi: 10.3389/fimmu.2025.1436311 39917306 PMC11799277

[18] Li J Xiang T Chen X Fu P. Neutrophil-percentage-to-albumin ratio is associated with chronic kidney disease: evidence from NHANES 2009-2018. PLoS ONE. (2024) 19:e0307466. doi: 10.1371/journal.pone.0307466 39102412 PMC11299806

[19] Wang Y Chen S Tian C Wang Q Yang Z Che W . Association of systemic immune biomarkers with metabolic dysfunction-associated steatotic liver disease: a cross-sectional study of NHANES 2007-2018. Front Nutr. (2024) 11:1415484. doi: 10.3389/fnut.2024.1415484 39296508 PMC11408230

[20] Johnson AF Lamontagne N Bhupathiraju SN Brown AG Eicher-Miller HA Fulgoni VL . Workshop summary: building an NHANES for the future. Am J Clin Nutr. (2024) 119:1075–81. doi: 10.1016/j.ajcnut.2024.02.001 38331096 PMC11181347

[21] Dai S Shu D Meng F Chen Y Wang J Liu X . Higher risk of sarcopenia in older adults with type 2 diabetes: NHANES 1999-2018. Obes Facts. (2023) 16:237–48. doi: 10.1159/000530241 37011596 PMC10826600

[22] Bossi P Delrio P Mascheroni A Zanetti M. The spectrum of malnutrition/cachexia/sarcopenia in oncology according to different cancer types and settings: a narrative review. Nutrients. (2021) 13:1980. doi: 10.3390/nu13061980 34207529 PMC8226689

[23] Mousa N Salah M Elbaz S Elmetwalli A Elhammady A Abdelkader E . Neutrophil percentage-to-albumin ratio is a new diagnostic marker for spontaneous bacterial peritonitis: a prospective multicenter study. Gut Pathog. (2024) 16:18. doi: 10.1186/s13099-024-00610-2 38561807 PMC10985869

[24] Lan CC Su WL Yang MC Chen SY Wu YK. Predictive role of neutrophil-percentage-to-albumin, neutrophil-to-lymphocyte and eosinophil-to-lymphocyte ratios for mortality in patients with COPD: evidence from NHANES 2011-2018. Respirology. (2023) 28:1136–46. doi: 10.1111/resp.14589 37655985

[25] Batsis JA Mackenzie TA Jones JD Lopez-Jimenez F Bartels SJ. Sarcopenia, sarcopenic obesity and inflammation: results from the 1999-2004 national health and nutrition examination survey. Clin Nutr. (2016) 35:1472–83. doi: 10.1016/j.clnu.2016.03.028 27091774 PMC6432912

[26] Huang Q Wan J Nan W Li S He B Peng Z. Association between manganese exposure in heavy metals mixtures and the prevalence of sarcopenia in US adults from NHANES 2011-2018. J Hazard Mater. (2024) 464:133005. doi: 10.1016/j.jhazmat.2023.133005 37988867

[27] He R Ye Y Zhu Q Xie C. Association between non-high-density lipoprotein cholesterol to high-density lipoprotein cholesterol ratio and sarcopenia in individuals with cancer: a cross-sectional study. Lipids Health Dis. (2024) 23:217. doi: 10.1186/s12944-024-02205-x 39014376 PMC11251101

[28] Huang Z Peng W Zhao M Gao B Qian J Zhu S . Joint association of systemic inflammatory response index and sarcopenia with mortality among individuals with self-reported cancer. BMC Cancer. (2025) 25:267. doi: 10.1186/s12885-025-13653-8 39953396 PMC11829532

[29] Zeng QY Qin Y Shi Y Mu XY Huang SJ Yang YH . Systemic immune-inflammation index and all-cause and cause-specific mortality in sarcopenia: a study from national health and nutrition examination survey 1999-2018. Front Immunol. (2024) 15:1376544. doi: 10.3389/fimmu.2024.1376544 38638440 PMC11024272

[30] Aduse-Poku L Karanth SD Wheeler M Yang D Washington C Hong YR . Associations of total body fat mass and skeletal muscle index with all-cause and cancer-specific mortality in cancer survivors. Cancers. (2023) 15:1081. doi: 10.3390/cancers15041081 36831420 PMC9953880

[31] Barbosa-Silva TG Menezes AM Bielemann RM Malmstrom TK Gonzalez MC Grupo de. Estudos em Composição Corporal e Nutrição (COCONUT). Enhancing SARC-F: improving sarcopenia screening in the clinical practice. J Am Med Dir Assoc. (2016) 17:1136–41. doi: 10.1016/j.jamda.2016.08.004 27650212

[32] Bahat G Erdogan T Ilhan B. SARC-F and other screening tests for sarcopenia. Curr Opin Clin Nutr Metab Care. (2022) 25:37–42. doi: 10.1097/MCO.0000000000000801 34861669

[33] Malmstrom TK Morley JE. SARC-F: a simple questionnaire to rapidly diagnose sarcopenia. J Am Med Dir Assoc. (2013) 14:531–2. doi: 10.1016/j.jamda.2013.05.018 23810110

[34] Sun L Fu J Mu Z Duan X Chan P Xiu S. Association between body fat and sarcopenia in older adults with type 2 diabetes mellitus: a cross-sectional study. Front Endocrinol. (2023) 14:1094075. doi: 10.3389/fendo.2023.1094075 36777353 PMC9911832

[35] Kurkiewicz K Gasior M Szyguła-Jurkiewicz BE. Markers of malnutrition, inflammation, and tissue remodeling are associated with 1-year outcomes in patients with advanced heart failure. Pol Arch Intern Med. (2023) 133:16411. doi: 10.20452/pamw.16411 36633195

[36] Templeton AJ McNamara MG Šeruga B Vera-Badillo FE Aneja P Ocaña A . Prognostic role of neutrophil-to-lymphocyte ratio in solid tumors: a systematic review and meta-analysis. J Natl Cancer Inst. (2014) 106:dju124. doi: 10.1093/jnci/dju124 24875653

[37] Islam MM Satici MO Eroglu SE. Unraveling the clinical significance and prognostic value of the neutrophil-to-lymphocyte ratio, platelet-to-lymphocyte ratio, systemic immune-inflammation index, systemic inflammation response index, and delta neutrophil index: an extensive literature review. Turk J Emerg Med. (2024) 24:8–19. doi: 10.4103/tjem.tjem_198_23 38343523 PMC10852137

[38] Jiang X Xiao X Li H Gong Y Wang M Yang H . Oxidized galectin-1 in SLE fails to bind the inhibitory receptor VSTM1 and increases reactive oxygen species levels in neutrophils. Cell Mol Immunol. (2023) 20:1339–51. doi: 10.1038/s41423-023-01084-z 37737309 PMC10616122

[39] Cederholm T Barazzoni R Austin P Ballmer P Biolo G Bischoff SC . ESPEN guidelines on definitions and terminology of clinical nutrition. Clin Nutr. (2017) 36:49–64. doi: 10.1016/j.clnu.2016.09.004 27642056

[40] Pacifico J Geerlings MAJ Reijnierse EM Phassouliotis C Lim WK Maier AB. Prevalence of sarcopenia as a comorbid disease: a systematic review and meta-analysis. Exp Gerontol. (2020) 131:110801. doi: 10.1016/j.exger.2019.110801 31887347

[41] Papadopoulou SK. Sarcopenia: a contemporary health problem among older adult populations. Nutrients. (2020) 12:1293. doi: 10.3390/nu12051293 32370051 PMC7282252

[42] Wang L Liu L Liu X Yang L. The association between neutrophil percentage-to-albumin ratio (NPAR) and depression among US adults: a cross-sectional study. Sci Rep. (2024) 14:21880. doi: 10.1038/s41598-024-71488-y 39300155 PMC11413241

[43] Chew J Tay L Lim JP Leung BP Yeo A Yew S . Serum myostatin and IGF-1 as gender-specific biomarkers of frailty and low muscle mass in community-dwelling older adults. J Nutr Health Aging. (2019) 23:979–86. doi: 10.1007/s12603-019-1255-1 31781728 PMC12280681

