# Supplementary material for: Association between neutrophil percentage to albumin ratio and sarcopenia among cancer patients: evidence from both the Chinese and American cohorts
Source: Front Nutr. 2026 Jan 20;12:1709323. doi: 10.3389/fnut.2025.1709323 (PMC12864052; doi:10.3389/fnut.2025.1709323)
Supplement: Supplementary file 1 [file Supplementary_file_1.docx]

**Supplementary Table S1, Threshold effect analysis in Nhanes cohort（SARF.C criteria）**

| Scenario | Analysis Type | Indicator | OR(95%CI) | *P*-value |
| --- | --- | --- | --- | --- |
| Scenario I | Linear Effect | Overall Effect | 1.16 (1.09, 1.24) | ＜0.001 |
| Scenario II | Non-linear Effect | Threshold (K) | 14.9 | - |
| Scenario II | Non-linear Effect | Effect Below K | 1.04 (0.92, 1.17) | 0.521 |
| Scenario II | Non-linear Effect | Effect Above K | 1.26 (1.14, 1.40) | ＜0.001 |
| Scenario II | Non-linear Effect | Difference in Effects (2 vs. 1) | 1.21 (1.00, 1.46) | 0.044 |
| Scenario II | Non-linear Effect | Likelihood Ratio Test | - | 0.072 |

**Supplementary Table S2, Threshold effect analysis in Nhanes cohort（EWGSOP criteria）**

| Scenario | Analysis Type | Indicator | OR(95%CI) | *P*-value |
| --- | --- | --- | --- | --- |
| Scenario I | Linear Effect | Overall Effect | 1.06 (1.01, 1.10) | 0.0146 |
| Scenario II | Non-linear Effect | Threshold (K) | 13.2 | - |
| Scenario II | Non-linear Effect | Effect Below K | 0.95 (0.86, 1.04) | 0.260 |
| Scenario II | Non-linear Effect | Effect Above K | 1.11 (1.05, 1.18) | ＜0.001 |
| Scenario II | Non-linear Effect | Difference in Effects (2 vs. 1) | 1.18 (1.03, 1.34) | 0.015 |
| Scenario II | Non-linear Effect | Likelihood Ratio Test | - | 0.031 |

**Supplementary Table S3, Threshold effect analysis in Hospital cohort（AWGS2019 criteria）**

| Scenario | Analysis Type | Indicator | OR(95%CI) | *P*-value |
| --- | --- | --- | --- | --- |
| Scenario I | Linear Effect | Overall Effect | 1.06 (1.01, 1.11) | 0.021 |
| Scenario II | Non-linear Effect | Threshold (K) | 12.4 | - |
| Scenario II | Non-linear Effect | Effect Below K | 1.23 (1.02, 1.48) | 0.033 |
| Scenario II | Non-linear Effect | Effect Above K | 1.02 (0.96, 1.09) | 0.495 |
| Scenario II | Non-linear Effect | Difference in Effects (2 vs. 1) | 0.83 (0.67, 1.04) | 0.107 |
| Scenario II | Non-linear Effect | Likelihood Ratio Test | - | 0.150 |
